# Multi-functional bioactive secondary metabolites derived from endophytic fungi of marine algal origin

**DOI:** 10.1016/j.crmicr.2021.100037

**Published:** 2021-05-12

**Authors:** Harikrishnan M, Saipriya P. P, Prabha Prakash, C. Jayabaskaran, Sarita G. Bhat

**Affiliations:** a Department of Biotechnology, Cochin University of Science and Technology, Cochin, Kerala, India; b Department of Biochemistry, Indian Institute of Science, Bangalore, Karnataka, India

**Keywords:** Endophytic fungi, marine algae, antioxidant, antimicrobial, cytotoxic, GC-MS

## Abstract

Endophytic fungi from marine macroalgae are endowed with various pharmacologically active metabolites. This study mined, identified and screened endophytic fungi (EF) isolated from marine algae from the west coast of South India for screening anticancer, antioxidant and antimicrobial secondary metabolite producers. Five EF isolated from sampled marine algae were identified using morphology and ITS based identification as *Grammothele fuligo, Rigidoporus vinctus, Cystobasidium minutum, Candida railenensis* and *Pichia kudriavzevii.* After culturing 20 days on PDA medium, the ethyl acetate extracts of *Cystobasidium minutum* showed potent antimicrobial activity against *P. aeruginosa* with an IC_50_ value of 458.7 ± 1.021 µg/mL whereas *Pichia kudriavzevii* extracts showed promising antioxidant activity (IC_50_ value of 65.78 ± 1.082 µg/mL, 38.74 ± 1.040 µg/mL and 32.01 ± 1.018 µg/mL for DPPH assay, ABTS assay and FRAP assay respectively) and high cytotoxic activity against MG63 cell line (IC_50_ = 145.1 ± 1.086 µg/mL, no activity against U87 cells). The phytochemical screening of the extracts unveiled the existence of diverse groups of secondary metabolites. Further, Gas Chromatography Mass Spectroscopy (GC-MS) analysis of the extract revealed the presence of compounds that are known to be antibacterial, antioxidant and cytotoxic. These results indicate that marine derived endophytes could be potent sources for multi-functional bioactive compounds and may find prospective application in pharmaceutical industry.

## Highlights

• Marine algae of Kerala coastline are rich in endophytic fungal biodiversity
• Fungal isolates displayed promising antibacterial, antioxidant and cytotoxic activity
• Gas Chromatography based identification assisted in further metabolite discovery
• Various non-therapeutic applications reported earlier also adds to the significance.

## Graphical abstract

**Figure:**
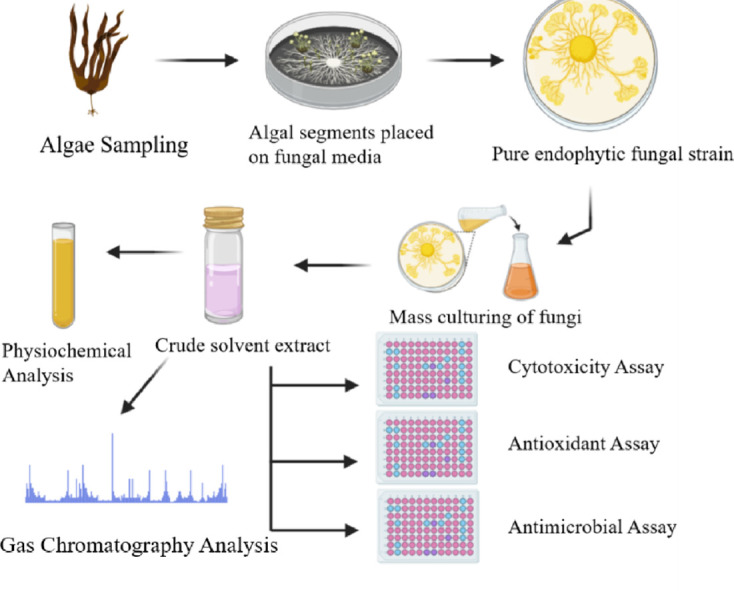

## 1 Introduction

Marine habitat with inorganic substrates (soil, sediments, artificial substrates and water column), marine plants (algae, mangrove plants and driftwood), marine invertebrates (especially sponges but also includes ascidians, corals, bivalves and holothurians), and vertebrates (mainly fish) are prolific sources for finding fungi. Marine-derived endophytic fungi, colonize internal tissues without causing palpable damage to their hosts, and are prolific sources of bioactive natural products with unique structures and potent pharmaceutical actions (Bugni and Ireland, 2004; Hasan *et al*., 2015; Deshmukh *et al*., 2018). Several novel bioactive compounds with varied biological properties, including anticancer, antibiotic, antiviral, and antioxidant have been isolated from marine algicolous endophytic fungi (Ashok *et al*., 2015, Flewelling *et al*., 2015; Rateb and Ebel, 2011). Indole derivatives produced by algicolous *Paecilomyces variotii* were found to be cytotoxic (Deshmukh *et al*., 2018). The crude extracts of marine alga (*B. tenella*) derived *P. decaturense* and *P. waksmanii*, exhibited potent antimicrobial and anticancer activity (De Felício *et al*., 2015).

Indian coastline (especially south Indian) with numerous rocky beaches presents a huge opportunity for finding wide variety of marine algae. A study revealed that 844 species of marine algae with primarily 216 species of Chlorophyta, 191 species of Phaeophyta, 434 species of Rhodophyta and 3 species of Xanthophyta have been reported from India (Ushakiran *et al*., 2017). The south Indian coastline, especially Kerala has a vast diversity of marine algae. In 1988, Chennubhotla and co-workers conducted the first extensive research assessment survey along the Kerala coastline bringing out the details of all the economically available important resources including marine algae and since then, novel bioactive secondary metabolites, especially from these marine microorganisms have always been in the limelight. Even though, Kerala is known to harbor a wide variety of algal flora, there are only few reports on the screening of marine algicolous endophytic fungi for various biological activities. The present study was conducted to isolate endophytic fungi from marine algae residing along the Kerala coastline and to evaluate its antimicrobial, antioxidant and cytotoxic activities.

## 2 Materials and methods

### 2.1 Sample preparation

Algal samplings were conducted at Munambam, Cochin, Kerala, India (10.1772° N, 76.1655° E), and Varkala beach, Trivandrum, Kerala, India (8.7356° N, 76.7032° E) using sterile gloves during low tide. Care was taken to avoid dead or decaying part of algae and collect only healthy and fresh algal leaves. Marine algae were rinsed in sterile sea water and immersed in 70% ethanol for 60-120 seconds, before being sealed in plastic bags containing sterile sea water. The samples were then stored in ice and transported to the laboratory for further investigation.

### 2.2 Isolation of endophytic fungi from marine algae

In the laboratory, they were washed thoroughly in running tap water to remove debris. For eliminating epiphytic fungi, surface sterilization was performed by sequentially immersing in 70% ethanol for 60-120s, then in aqueous sodium hypochlorite (4% available chlorine) for 3 min, followed by a wash with 70% ethanol for 5-10 s. Lastly, the algal tissues were rinsed thrice in sterile distilled water and dried on sterile paper towel.

The algae were chopped finely (approx. 2 × 2 mm) using sterile scalpel and placed on Potato dextrose agar (PDA) (HiMedia, Mumbai, India) (supplemented with chloramphenicol (200 mg/L), such that the cut edges contacted the medium. Plates were incubated at 25°C–27°C for 5–7 days with regular monitoring. Actively growing fungal tips emerging from algal fragments were subsequently sub cultured on fresh PDA for identification and enumeration (Kjer *et al*., 2010). For preservation, all the isolated endophytic fungi were maintained on PDA slants layered with glycerol (15%, v/v). The pure culture of endophytic fungus was then sub-cultured on PDA without any antibiotic and incubated for 20 days at 25°C prior to extraction.

### 2.3 Taxonomic and molecular identification of algae and endophytic fungi

Taxonomic identification of algae involved analysing the morphology and the ecology of sample. For isolating fungal genomic DNA, protocol of Gupta *et al.,* 2013 was followed. Briefly, fungal cell cultures were grown in potato dextrose broth for 72h at 30°C on a shaker (Orbitek, Scigenics, India) at 150rpm. Biomass was pelleted by centrifugation at 13000 rpm for 5 min (Sigma, 3K30, Germany). Pellet was washed with distilled water followed by the addition of extraction buffer (200 mM Tris-HCl, 20 mM EDTA and 1 % SDS made to pH 8.5). 2.5M sodium acetate was added (vortex for complete mixing), and the mixture was kept at -20°C for 10-15 min followed by centrifugation (10,000 rpm, 4°C, 10 min.). Chilled isopropanol was added to the supernatant, centrifuged and the pellet was washed with ethanol, air dried and dissolved in 30 μL TE buffer. Quality of DNA was analyzed using Biospec-nano spectrophotometer (Shimadzu, Japan) as per Sambrook (Sambrook, 1989).

ITS region of fungal DNA was amplified using primers (ITS 1 primer: TCCGTAGGTGAACCTGCGG and ITS 4 primer: TCCTCCGCTTGATATGC) (White *et al*., 1990). The amplified product was subjected to electrophoresis using 0.8% agarose gel prepared in 1X TAE buffer (Lee *et al.,* 2012). The gel was stained with ethidium bromide (0.5 mg/mL) for 20 minutes and the image captured using Gel Documentation system (Syngene, UK). Furthermore, the amplified DNA was purified and sequenced by ABI XL DNA Analyzer, using the big dye terminator kit (Applied Biosystems, USA) at SciGenom, Cochin, India. NCBI BLAST N program was used to compare the sequence available in NCBI Gen Bank (Altschul *et al*., 1990).

Phylogenetic analysis was performed using MEGA version 7.0 (Kumar *et al*., 2016). NJ trees were constructed based on the total character differences and bootstrap values were calculated from 1,000 replications. Clustering using the neighbour-joining were determined using bootstrap values based on 1,000 replicates (Saitou and Nei, 1987). The phylogenetic tree was drawn with branch lengths in the units of the evolutionary distances which were computed using the Maximum Composite Likelihood method (Tamura *et al*., 2004).

### 2.4 Extraction of secondary metabolites

20-day pure culture of endophytic fungus on PDA was extracted thrice with equal volume of Ethyl Acetate for 24 h and filtered. The ethyl acetate extract (EtOAc extract) was evaporated using rotary evaporator (IKA Digital Rotary Evaporator, Sigma-Aldrich, USA) to obtain concentrated extract (Kjer *et al*., 2010).

### 2.5 Phytochemical screening of extract

Phytochemical screening for all EtOAc extracts was conducted by the standard protocol described by Tiwari *et al*., 2011. This method aims for qualitative identification of alkaloid, phenolic, terpenoid and steroid compounds in the extract. Briefly, 0.5 mL of diluted hydrochloric acid (10%) was added to 1 mL extract (1 mg/mL) and then filtered. 3-4 drops of Mayer's reagent (3 mL of potassium iodide solution mixed with 2 ml mercuric chloride solution) was added to test for alkaloids; yellow colour of the filtrate confirms the presence of alkaloids. Alkaloids precipitate out in presence of Mayer's reagent forming a creamy or yellow precipitate.

Similarly, 0.5 mL of diluted hydrochloric acid (10%) was added to 1 mL extract (1 mg/mL) and filtered. Ferric chloride solution (3-4 drops) was added and the change in colour of filtrate to Blue will confirm the presence of phenolic compounds. This is due to the formation of a bonding complex by ferric chloride and phenol which enables the electrons to get excited by the wavelengths of light. For steroid and terpenoid detection, chloroform was added to 1mL of extract (1 mg/mL) and then filtered. Addition of 1-2 drops of acetic anhydride (99%) and concentrated sulphuric acid to the filtrate, followed by shaking yields a blue or purple colour indicating the presence of steroid, while red colour indicates the presence of terpenoid. Terpenoids and steroids form chromogens with acidic solution of acetic anhydride (Tiwari *et al*., 2011).

### 2.6 Determination of antibacterial activity of fungal crude extracts

Antibacterial activity of the EF EtOAc extracts was tested against both Gram-positive and Gram-negative bacterial pathogens such as *Bacillus pumilus* (NCIM2189), *Staphylococcus aureus* (NCIM2147)*, Escherichia coli* (NCIM2343) and *Pseudomonas aeruginosa* (NCIM2863) using agar disk diffusion method (Bauer *et al*., 1966). Briefly, the test organisms were grown to OD_600_ =1 at 37°C and spread on Muller Hinton agar (MHA) plates. Pre-sterilized disks with fungal crude extract (500 µg/mL) were placed on MHA agar plates with the test organisms and incubated at 37°C for 24 h. The presence of zone of clearance around the disk was used as an indicator of (antibacterial) bioactivity. Ampicillin standard disk (HiMedia, India) and Ciprofloxacin standard disk (HiMedia, India) served as positive control, while DMSO (HiMedia, India) served as negative control respectively. All testing was repeated thrice for statistical evaluations.

Antimicrobial IC_50_ was determined by micro broth dilution assay as described by Wariso and Ebong, 1996 with minor modification. One millilitre of sterilized Muller Hinton Broth (MHB) was dispensed into the test tubes labelled from 1 to 5 using sterile syringe and needle. A stock of MHB containing 1000 µg/mL of crude extract was prepared and diluted twofold for five times in sterile tubes. Each tube was inoculated with equal volume of overnight growth culture of test organisms, incubated at 37°C for 24 h and examined for growth. In addition to negative control (Dimethyl sulfoxide), one tube each for control of medium sterility and viability of organism was included. Final concentration of the extract in each test tube numbered 1-5, after dilution was 1000, 500, 250, 125, 75.5, 37.75 µg/mL respectively (Wariso and Ebong, 1996). Absorbance data were converted into % cell inhibition according to the following equation:

$$\text{Cell}\;\text{inhibition}\;=\;\left(\frac{Abs.\;value\;of\;control-\;Abs.\;value\;of\;sample}{Abs\;value\;of\;control}\right)x\;100\%$$

Half maximal inhibitory concentration (IC_50_) was calculated from the dose-response curve.

### 2.7 Free radical 1,1-diphenyl-2-picryl-hydrazyl (DPPH)-scavenging test

DPPH free radical scavenging potential of fungal EtOAc extract was determined as per protocol explained by Brand-Williams, 1995 with slight modifications. This method is based on the reduction of purple coloured DPPH to a colourless solution due to the donation of electrons to DPPH free radicals (Brand-Williams *et al*., 1995). DPPH stock solution was prepared by mixing 0.1 mM DPPH in 99% ethanol and standardised to 1.9 ± 0.02 OD at 515 nm using UV- Vis Spectrophometer (Shimadzu, Japan). Subsequently 100 μL of the crude at concentrations ranging from 6.25 to 100 μg/mL and standard ascorbic acid (0.1 mg/mL) were mixed with 0.1 mL DPPH solution. Absorbance was measured at 515 nm after 30 min of incubation at room temperature. Percentage of the DPPH radical scavenging was calculated using the formula.

$$\begin{array}{ccc} & & \text{Scavenging}\;\text{Activity}\;\left(\%\right)\\ & & =1-\left(\frac{Abs.\;value\;of\;positive\;control-\;Abs.\;value\;of\;sample}{Abs\;value\;of\;positive\;control}\right)x\;100\% \end{array}$$

IC_50_ was calculated from the dose-response curve.

### 2.8 [2, 2’-azinobis-(3-ethylbenzothiazoline-6-sulfonic acid)] (ABTS) radical-scavenging test

The ABTS radical-scavenging activities of different dilutions of crude extract were determined using the protocol described by van den Berg *et al*.,1999, with slight modification. Stock solution was prepared by dissolving 7.4 mM ABTS and 2.6 mM potassium persulfate in Milli-Q water. After 16 h, phosphate buffered saline (PBS) pH 7.4 was added to concentrated ABTS stock solution to record an absorbance between 1.0 and 1.2 at 734 nm. Subsequently, 10 μL of the extract at concentrations ranging from 6.25 to 100 μg/mL were mixed with 990 μL of the ABTS radical solution and the absorbance was measured at 734 nm (Berg and M, 1999). Percentage of the ABTS radical scavenging was calculated using the formula.

$$\begin{array}{ccc} & & \text{Scavenging}\;\text{Activity}\;\left(\%\right)\\ & & =1-\left(\frac{Abs.\;value\;of\;positive\;control-\;Abs.\;value\;of\;sample}{Abs\;value\;of\;positive\;control}\right)x\;100\% \end{array}$$

IC_50_ values for the crude extracts were calculated from the dose-response curve.

### 2.9 Free radical scavenging assay by FRAP method

The total antioxidant potential or ferric reducing potential of the EtOAc extract was determined by the ferric reducing antioxidant power (FRAP) assay of Benzie and Strain, 1996. FRAP assay determines the reducing potential of an antioxidant reacting with a ferric tripyridyltriazine (Fe^3+^-TPTZ) complex and then producing a coloured ferrous tripyridyltriazine (Fe^2+^-TPTZ) by donating a hydrogen atom. At a pH of about 3.6, blue coloured Fe^2+^-TPTZ is reduced to Fe^3+^-TPTZ, which has an absorbance at 593 nm. In ABTS assay, the radical cation formed is blue in colour, which is reduced by the antioxidant generating a colourless solution (Rajurkar *et al*., 2011). A solution of 10 mmol/L 2,4,6-Tripyridyl-S-triazine (TPTZ) in 40 mmol/L HCl and 12 mmol/L ferric chloride was diluted in 300 mmol/L sodium acetate buffer (pH 3.6) at a ratio of 1:1:10. Crude extracts of concentrations ranging from 6.25 to 100 μg/mL were added to 3 mL FRAP solution, and allowed to react for 90 min at 37°C followed by measuring the absorbance at 593nm (Benzie and Strain, 1996). Percentage of the radical scavenging was calculated using the formula,

$$\begin{array}{ccc} & & \text{Scavenging}\;\text{Activity}\;\left(\%\right)\\ & & =1-\left(\frac{Abs.\;value\;of\;positive\;control-\;Abs.\;value\;of\;sample}{Abs\;value\;of\;positive\;control}\right)x\;100\% \end{array}$$

IC_50_ values for the crude extracts were calculated from the dose-response curve.

### 2.10 Human cancer cell lines and culture conditions

MG63 (Human osteosarcoma) and U87 (Human glioblastoma) cells were procured from National Centre for Cell Sciences (NCCS), Pune, cultured in Dulbecco's modified Eagle's medium (DMEM), supplemented with 10% Fetal Bovine Serum (FBS), 100 U/mL penicillin and 100 U/mL of streptomycin in a 5% CO_2_ atmosphere at 37°C.

### 2.11 In vitro cytotoxic activity of crude fungal extracts using MTT [3-(4, 5-Dimethylthiazol-2-yl)-2, 5 Diphenyltetrazolium Bromide] assay

The cytotoxic effects of fungal crude extracts on MG63 and U87 cell lines was assayed using MTT assay (Mosmann, 1983). Briefly, the cells were seeded in 96 well plates at a density of 1 × 10^4^ cells/well for 24 h. Subsequently, the fungal crude extracts in 0.4 % DMSO were added at a concentration of 500 μg/mL and incubated for 48 h at 37°C in an atmosphere of 5% CO_2_. Post incubation, MTT reagent (5 mg/mL in phosphate-buffered saline) was added followed by 4h dark incubation. The blue MTT formazan precipitate formed was solubilized in DMSO and measured spectrophotometrically at 570 nm in an ELISA plate reader scanning spectrophotometer (Tecan Microplate Reader, Switzerland). Cells grown in culture media alone or with appropriate concentrations of DMSO were used as controls, and paclitaxel was used as the positive control. Further, those compounds which exhibited potent cytotoxic activity, were subjected to MTT assay against the respective cell lines with concentrations ranging from 37.75 to 500 μg/mL to evaluate the half maximal inhibitory concentration. DMSO and Paclitaxel (0 – 1 μg/mL) was used as control. Absorbance data were converted into % cell inhibition according to the following equation:

$$\text{Cell}\;\text{inhibition}=\left(\frac{Abs.\;value\;of\;control-\;Abs.\;value\;of\;sample}{Abs\;value\;of\;control}\right)x\;100\%$$

The concentration required to produce half maximal inhibition (IC_50_) was then calculated from the dose-response curve.

### 2.12 Gas chromatography-mass spectrometry analysis

The crude fungal EtOAc extract was subjected to GC MS analysis to identify various bioactive compounds. The extract was analysed in GC Clarus 500 Perkin Elmer using Turbo mass 5.2 software equipped with mass detector Turbo mass gold Perkin Elmer. A 2µL sample volume was introduced via an all glass injector working in the split mode, with He as the carrier gas with a linear velocity of 32 cm/s. The HP-5 fused silica capillary column (Length – 30 m; Film thickness- 25 µm I.D - 0.2 mm) was used and the identification of components was accomplished using library searches in NIST version 2005 (Nameirakpam and Singh, 2013).

### 2.13 Statistical analysis

Each sample analysis was performed in triplicate. All of the presented results are the means (±standard error) of at least three independent experiments. Statistical analysis was performed by R software (“R Core Team (2020). R: A language and environment for statistical computing. R Foundation for Statistical Computing, Vienna, Austria.,” 2020) (Hothorn *et al*., 2008). For antimicrobial assay, antioxidant assay and cytotoxicity assay, statistical significance was evaluated using Analysis of Variance (ANOVA) followed by Dunnets's multiple comparison test, while for activity screening using MTT, Tukey's pairwise comparison test was performed (p < 0.05). It is to be noted that Tukey's multiple comparison test was used only if the control group is absent. The correlation between antioxidant and cytotoxic activity was assessed by calculating Pearson's coefficient r and its statistical reliability using GraphPad Prism version 8.0.0 for Windows, (GraphPad Software, San Diego, California USA, www.graphpad.com). Correlation was considered as very strong when r = 0.90 – 0.99 (positive) or −0.99 – (−0.90) (negative); strong when r = 0.70 – 0.89 (positive) or −0.89 – (−0.70) (negative); and moderate when r = 0.40 – 0.69 (positive) or −0.69 – (−0.40) (negative). Student's t-test was used for statistical comparison and the level of statistical significance was set at p < 0.05.

## 3 Results

### 3.1 Identification of sampled algae and isolated endophytic fungi

Sampling along Munambam and Varkala coastline yielded four green and one brown algae (Figure S1). Two green algae and one brown alga collected from Munambam while two green algae from Varkala beach (Figure S2A) were identified using morphological features and the geolocation of the sampling also played an important role in taxonomic identification. Taxonomically, the algal samples GA2, GA4 BA2, GAAB and GA21 were identified as belonging to genus *Enteromorpha, Rhizoclonium, Undaria, Ulva* and *Chaetomorpha* respectively (Table 1). The endophytic fungal isolates obtained from the collected algae GA2, GA4, BA2, GAAB and GA21 were labelled as BT-GA2, BT-GA421, BT-BA212, BT-GAAB1 and BT-GA211 respectively. Macroscopically, isolates BT-GA421, BT-GAAB1 and BT-GA211 were observed to have yeast-like growth, while BT-BA212 and BT-GA2 had fur-like growth (Figure S2B).

**Table 1:** Summary of sampling sites, algae samples and the identified endophytic fungi isolates.

Geological Information (Latitude & Longitude)	Sampling Site	Algae Isolates obtained	Morphological features	Taxonomic Identification	Fungal Isolates obtained	Growth Characteristics	Molecular Identification	GenBank Accession Number
10.1772° N, 76.1655° E	Munambam Beach (sandy), Cochin, Kerala, India	Green algae – **GA2, GA4**	**GA2** – Green inflated tubular unbranched fronds	***Enteromorpha*** (Family – Ulvaceae)	**BT-GA2**	white fur growth	***Rigidoporus vinctus***	MK920214
			**GA4** – Small sized green bush like filamentous formations	***Rhizoclonium*** (Family – Capsosiphonaceae)	**BT-GA421**	Pale white coloured yeast like growth	***Cystobasidium minutum***	MT372471
		Brown algae – **BA2**	**BA2** – Slight brown coloured algae with frilly sporophylls	***Undaria*** (Family – Alariaceae)	**BT-BA212**	white fur like growth	***Grammothele fuligo***	MT372472
8.7356° N, 76.7032° E	Varkala Beach (rocky), Trivandrum, Kerala, India	Green algae – **GAAB**	**BT-GAAB1** – Membranous, thin, delicate short sheet like morphology	***Ulva*** (Family – Ulvaceae)	**BT-GAAB1**	white, smooth yeast like growth	***Candida railenensis***	MK920215
		Brown algae – **GA21**	**GA21** – Filamentous loosely entangled mass like morphology	***Chaetomorpha*** (Family – Cladophoraceae)	**BT-GA211**	yeast like growth and alcohol production in media	***Pichia Kudriavzevii***	MT372473

**Fig. 1:**
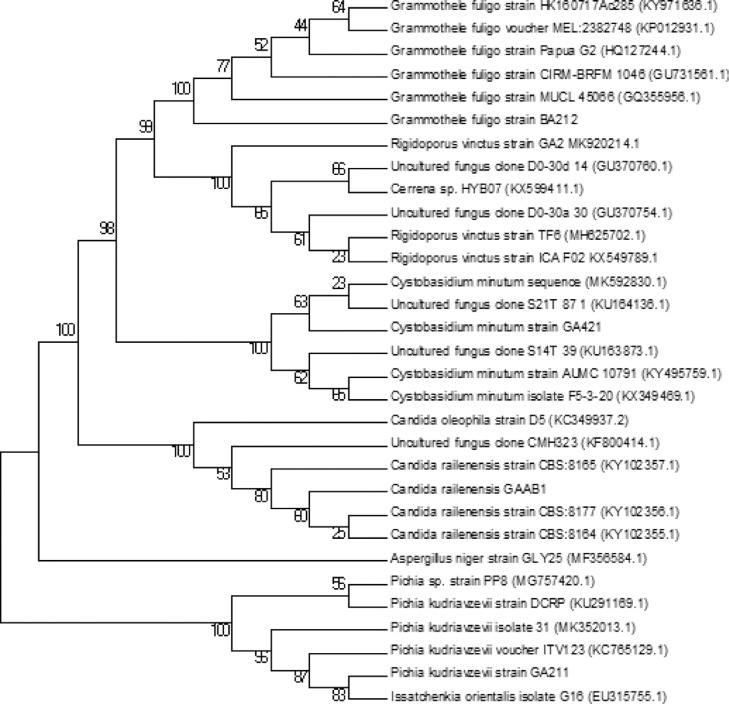
Phylogenetic Tree of fungal isolates constructed using Neighbour-Joining method in MEGA6. BT-BA212, BT-GA2, BT-GA421, BT-GAAB1, BT-GA211 were identified as Grammothele fuligo, Rigidoporus vinctus, Cystobasidium minutum, Candida railenensis, Pichia Kudriavzevii respectively. The tree is drawn to scale, with branch lengths in the same units as those of the evolutionary distances used to infer the phylogenetic tree. The evolutionary distances were computed using the Tajima-Nei method and are in the units of the number of base substitutions per site.

For molecular identification, following DNA isolation, a 600bp amplicon was obtained after PCR amplification of 18S rRNA gene of the EF isolates. After sequencing, the amplicon of BT-BA212 showed 99% identity to 18S rRNA gene of *Grammothele fuligo* (KC015263.1). Phylogenetic tree constructed using MEGA7 also depicted cladding of the sample with other *Grammothele* samples, thereby confirming the identity of the isolate (Figure 1). The sequence was submitted to GenBank and accession number was obtained (MT372472). Similarly, BT-GA2, BT-GA421, BT-GAAB1 and BT-GA211 were identified as *Rigidoporus vinctus, Cystobasidium minutum, Candida railenensis* and *Pichia Kudriavzevii* respectively and accession numbers obtained (Table 1).

Two strategic locations along southern and mid regions of Kerala coastline were selected for marine algal sampling as there was not much investigations conducted on the endophytic fungi obtained from these locations. A rich diversity and luxuriant growth of marine algae along Munambam and Varkala coastline due to the presence of rocks, bedrocks and cliffs was reported (Yadav *et al*., 2015). It may be noted that *Enteromorpha sp., Rhizoclonium sp.* and *Ulva sp*. are green algae while *Chaetomorpha sp.* and *Undaria sp.* are brown algae. Except *Undaria sp.* these algae have been isolated from various Indian coastal locations (Ghosh and Keshri, 2010; Paul, 2013; Raghukumar C, 2008). A review on economically important seaweeds of Kerala coast has highlighted the frequent occurrence of *Ulva sp*. and *Enteromorpha sp*. (Yadav *et al*., 2015). All isolated macroalgal species have already been studied apropos their associated fungal communities (Godinho *et al*., 2013).

It is interesting to note that the EF from algae sampled along Munambam coastline belonged to Basidiomycota, while those obtained from algae sampled along Varkala coastline belonged to Ascomycota. It is the first of its kind as Fungi belonging to phylum Basidiomycota are very rarely reported in marine habitats as they find it difficult to adapt to marine conditions due to their large putrescent fruit bodies and spore-discharge mechanism, but there are reports of occurrence of basidiomycetes from Kerala coastline with a smaller sporocarp than their terrestrial counterparts (Raveendran K and Manimohan, 2007). While reporting these fungi, the identification method utilized plays an essential role in accepting the fungi according to the latest International Code of Nomenclature for algae, fungi, and plants. Morphological characters can be misleading so morphology-based identification cannot be relied on. Use of internal transcribed spacer region (ITS) as an official DNA barcode marker for fungi has greatly benefitted fungal research, but presence of intragenomic ITS variation makes it more challenging. However, protein coding regions such as beta tubulin are used to augment ITS based identification as the intron region present in between these coding regions evolve at a faster rate as compared to ITS (Raja *et al*., 2017). There are only few basidiomycetes documented from marine habitats whereas ascomycota is the most dominant group in marine mycology, which is due to their inbuilt ability to withstand fluctuating saline conditions along with small sporocarps. The phylogenetic tree of *Rigidoporus vinctus* and *Pichia Kudriavzevii* reveals that their identification is a little bit challenging, hence a follow-up study needs to be conducted using beta tubulin based identification along with incorporating the same for future fungal identification protocols. Detailed information on endophytic marine fungi of the Kerala coast is scarce. Although some occasional collections of endophytic fungi from some locations in the Kerala coast have been made, a thorough and systematic study on marine fungi of this region are conspicuous by their absence (Raveendran K and Manimohan, 2007).

### 3.2 Phytochemical screening of fungal crude extracts

Phytochemical screening of crude extracts identified the presence of alkaloid, phenolic, terpenoid and steroid compounds (Table S1). All extracts showed the presence of alkaloids, except *Candida railenensis* BT-GAAB1, while all except *Rigidoporus vinctus* BT-GA2 exhibited the presence of phenolic compounds. But, it has been observed that only *Rigidoporus vinctus* BT-GA2 and *Candida railenensis* BT-GAAB1 tested positive for terpenoids. Similarly, only *Grammothele fuligo* BT-BA212 and *Rigidoporus vinctus* BT-GA2 showed the presence of steroids (Table S1).

### 3.3 Antibacterial activity

**Fig. 2:**
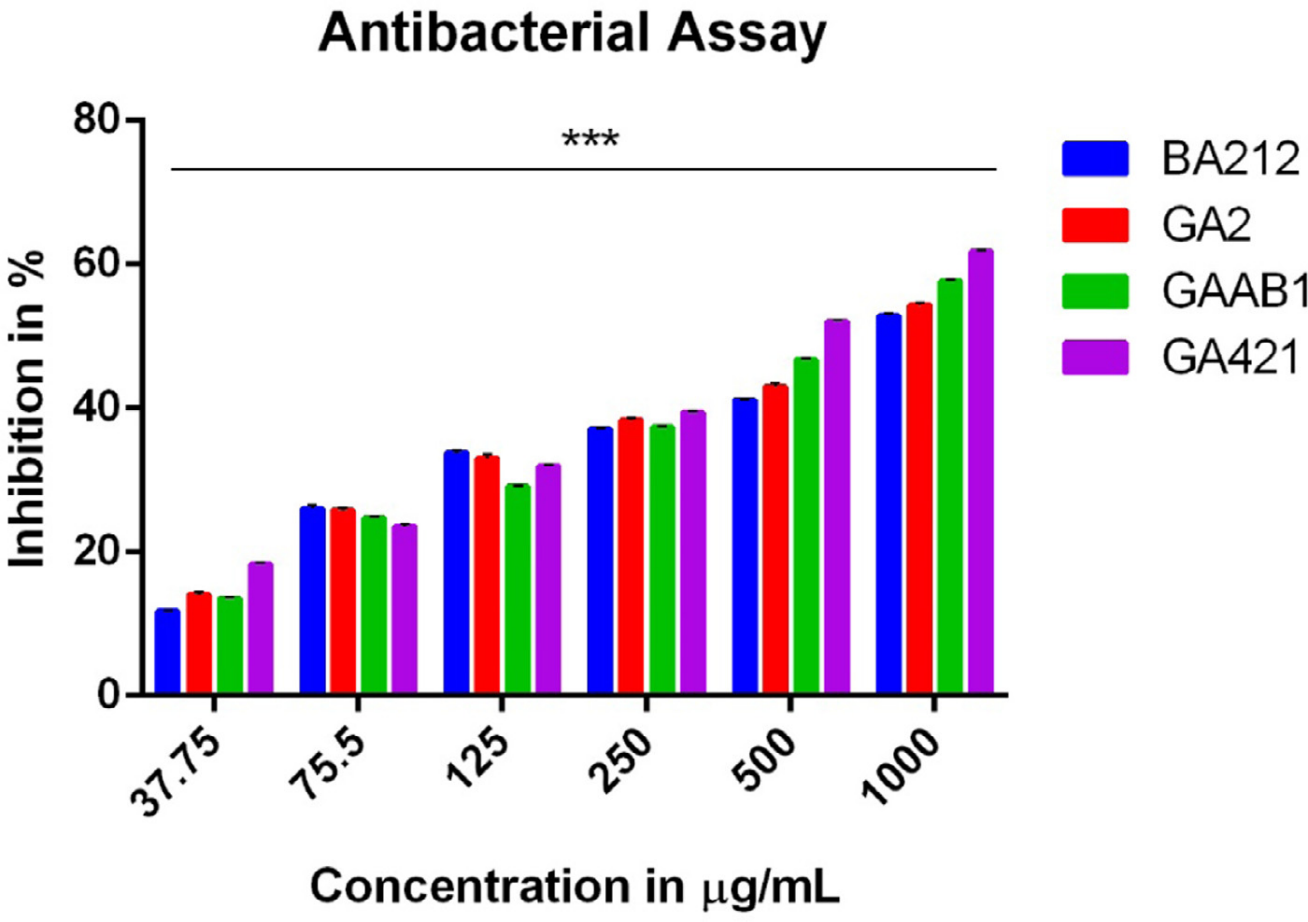
Antibacterial Assay of fungal crude extracts. Micro dilution assay was performed with different concentrations of fungal extracts. The results were presented as the mean % inhibition (± SE, n=3). Statistical significance is represented by ***, p < 0.001

Disk diffusion assay for antibacterial activity of the crude extracts against *P. aeruginosa, E. coli, S. aureus* and *B. pumilus* included ampicillin and ciprofloxacin as positive controls (Table S2). Surprisingly, all the extracts, except *Pichia kudriavzevii* BT-GA211, displayed potent activity only against *P. aeruginosa* and were ineffective against the other pathogens. Further, those crude extracts that displayed potent activity against *P. aeruginosa* were subjected to micro broth dilution assay*.* It was observed that at a concentration of 37.75 µg/mL, all the extracts showed an inhibition of 10-20% as compared to the control, while as the concentration increased to 1000 µg/mL, there was a steep increase in percentage of inhibition to 45-60%. At a concentration of 1000 µg/mL, the maximum percentage of inhibition was displayed by *Cystobasidium minutum* BT-GA421 (62%) followed by *Candida railenensis* BT-GAAB1 (58%), *Rigidoporus vinctus* BT-GA2 (54%) and *Grammothele fuligo* BT-BA212 (53%) (Figure 2). The analysis was done in triplicates and the results were statistically analysed using One-way ANOVA. All the extracts were significantly different compared to control with a ***p < 0.001. Moreover, IC_50_ was calculated for all the active crude extracts and interestingly, it was observed that lowest IC_50_ was observed in the crude extract of *Cystobasidium minutum* BT-GA421 (458.7 ± 1.021), while the highest was displayed by *Grammothele fuligo* BT-BA212 (793.2 ± 1.138) (Table 2). Dunnet's Multiple Comparison was also performed followed by ANOVA and all the crude extracts were significantly different between the treatment groups ***p < 0.001.

**Table 2:** Antibacterial activity assay of fungal crude extracts.

IC_50_ values in the Antibacterial activity assay of ethyl acetate extracts of fungal isolates	
IC_50_ (μg/mL)	
Isolates	*P. aeruginosa*
BT-BA212	793.2 ± 1.138
BT-GA2	709.7 ± 1.092
BT-GAAB1	594.2 ± 1.037
BT-GA421	458.7 ± 1.021

It was surprising to note that except *Grammothele fuligo* BT-BA212, all the fungi that exhibited potent antimicrobial activity were isolated from green algae and it has been reported that marine green algae are well known to produce antimicrobial compounds (Shannon and Abu-Ghannam, 2016). However, there are no previous reports of these fungi producing any antimicrobial compounds, so there is a high possibility of endophytes gaining the gene for antimicrobial compound production from their host by horizontal gene transfer.

### 3.4 Free radical scavenging activity

**Fig. 3:**
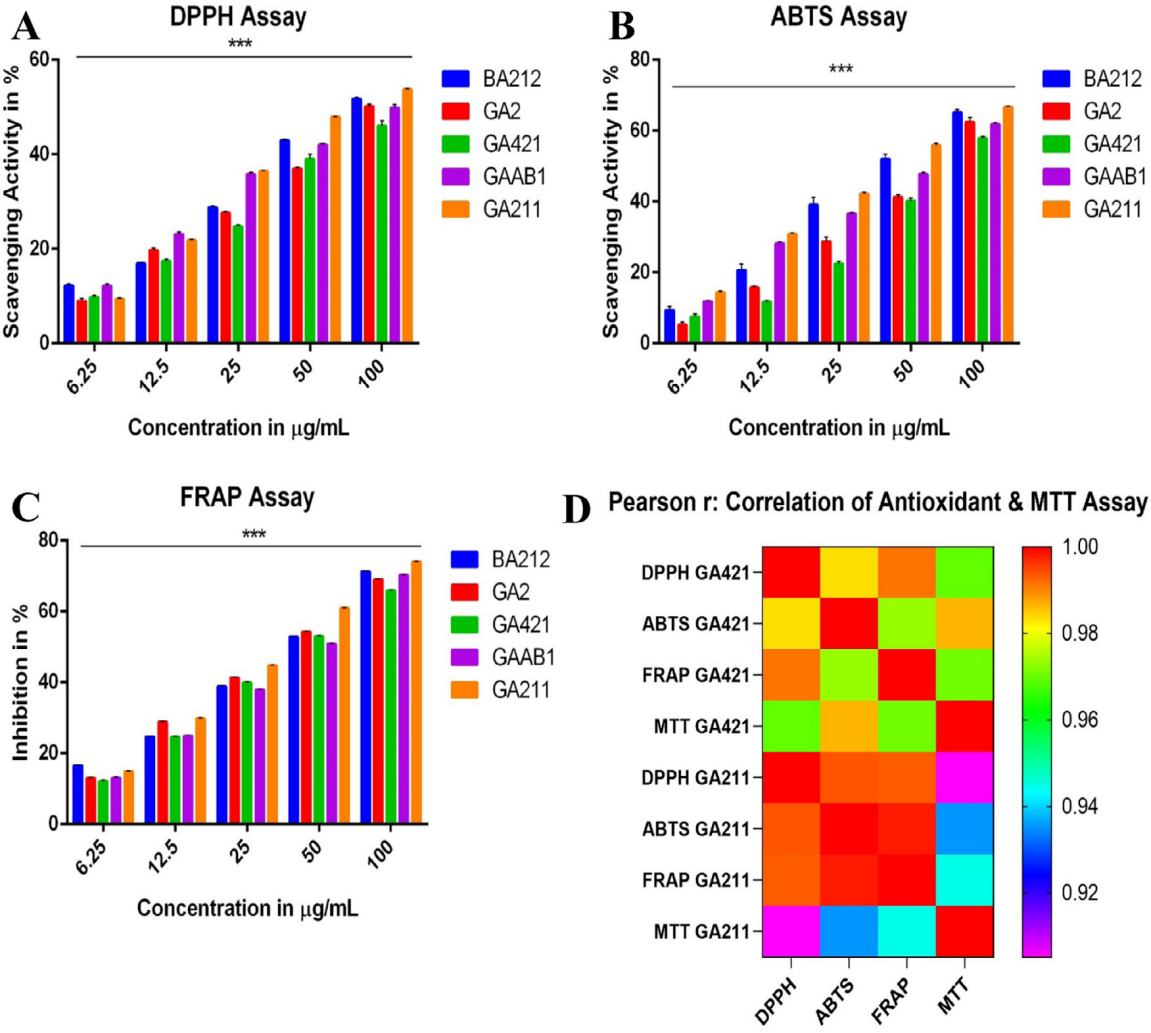
Antioxidant assay and Correlation Assessment using Pearson's coefficient r. A, B and C represents DPPH Assay, ABTS Assay and FRAP Assay respectively, using different concentration of crude extracts ranging 6.25 to 100 μg/mL. The results were presented as the mean % scavenging activity (± SE, n=3). Statistical significance is represented by ***, p < 0.001. D, Correlation of antioxidant and MTT Assay was assessed using Pearson's r coefficient. The results are presented on a scale of 0.9 to 1.0 based on its ‘r’ value and all the results were statistically relevant with p < 0.01.

The free radical scavenging activity of fungal crude extracts were studied using three methods namely, DPPH assay, ABTS assay and FRAP assay, using ascorbic acid as the standard. For DPPH Assay, as the concentration increased from 6.25 µg/mL to 100 µg/mL, the percentage scavenging activity varied from 10% to 50% (approx.) as compared to control, for all five crude extracts. At the highest concentration, which is 100 µg/mL, the highest scavenging activity of 54% was shown by *Pichia kudriavzevii* BT-GA211, while the lowest (46%) was observed in *Cystobasidium minutum* BT-GA421. In addition, IC_50_ values were calculated, which was highest for *Cystobasidium minutum* BT-GA421 (111.9 ± 1.067) crude, while the lowest (65.78 ± 1.082) was observed in *Pichia kudriavzevii* BT-GA211 extract. Interestingly, IC_50_ value displayed by *Pichia kudriavzevii* BT-GA211 was comparable to Ascorbic acid, which showed an IC_50_ value of 43.23 ± 1.12 (Figure 3A).

Similarly, for ABTS assay, it was observed that at a concentration of 6.25 µg/mL, all five crude extracts showed approximately 15-20% scavenging activity as compared to control, while at a concentration of 100 µg/mL, a sharp increase in activity (60-70%) was observed. At the highest concentration, the maximum scavenging activity was displayed by *Pichia kudriavzevii* BT-GA211 with an activity of 67%, while the lowest was observed in *Cystobasidium minutum* BT-GA421 (58%). Further, IC_50_ values were calculated to estimate the half maximal inhibitory concentration and interestingly, it was unveiled that the lowest IC_50_ was observed in the extract of *Pichia kudriavzevii* BT-GA211 (38.74 ± 1.040), while the highest was displayed by *Cystobasidium minutum* BT-GA421 (74.11 ± 1.021). The maximum scavenging activity displayed by *Pichia kudriavzevii* BT-GA211 was comparable to ascorbic acid (29.02 ± 1.76) (Figure 3B).

Ferric ion reducing activity was assessed by FRAP assay and it was noted that at the highest concentration (100 µg/mL), maximum scavenging activity was displayed by *Pichia kudriavzevii* BT-GA211 with an activity of 74%, while the lowest was observed in *Cystobasidium minutum* BT-GA421 (66%). In general, the scavenging activity increased with increasing concentration from 6.25 µg/mL to 100 µg/mL. In addition, on analysing the IC_50_ values, it was exhibited that the lowest IC_50_ was observed in the extract of *Pichia kudriavzevii* BT-GA211 (32.01 ± 1.018), while the highest was displayed by *Grammothele fuligo* BT-BA212 (40.88 ± 1.020). Again, as observed in above two assays, the highest scavenging activity was displayed by *Pichia kudriavzevii* BT-GA211 and the IC_50_ was comparable to ascorbic acid (21.78 ± 1.17) (Figure 3C). Interestingly, it was observed that all the three assays produced similar activity profile with crude extract of *Pichia kudriavzevii* BT-GA211 exhibiting potent scavenging activity. Table 3 lists consolidated IC_50_ values exhibited by all the five fungi in the three assays (Table 3). For all the three analysis, One-way ANOVA was performed followed by Dunnet's Multiple Comparison. All the crude extracts were significantly different between the treatment groups ***p < 0.001.

**Table 3:** Free radical scavenging activity assay of crude fungal extracts.

IC_50_ values in the DPPH, ABTS, and OH radical-scavenging activity assay of ethyl acetate extracts of fungal isolates			
IC_50_ (μg/mL)			
Isolates	DPPH radical scavenging	ABTS radical scavenging	Ferric ion radical scavenging
BT-BA212	83.11 ± 1.043	47.08 ± 1.051	40.88 ± 1.020
BT-GA2	98.42 ± 1.046	64.05 ± 1.035	39.54 ± 1.031
BT-GA421	111.9 ± 1.067	74.11 ± 1.021	44.27 ± 1.028
BT-GAAB1	86.99 ± 1.092	53.03 ± 1.051	43.36 ± 1.023
BT-GA211	65.78 ± 1.082	38.74 ± 1.040	32.01 ± 1.018
Ascorbic Acid	43.23 ± 1.12	29.02 ± 1.76	21.78 ± 1.17

Based on the calculated Pearson coefficient, all of the fungal crude extracts exhibited a very strong correlation (r = 0.9 – 0.99) between the three free radical scavenging properties. *Cystobasidium minutum* BT-GA421 exhibited a highest correlation between DPPH assay and ABTS assay with r value of 0.991, while *Pichia kudriavzevii* BT-GA211 exhibited the strongest correlation between FRAP assay and ABTS assay with r value of 0.998 (Figure 3D). Also, the correlations were found to be statistically significant (*p* > 0.01).

In case of *P. Kudriavzevii*, the high antioxidant activity is in accordance with the GC-MS analysis, which revealed the presence of Butylated Hydroxytoluene, a potent antioxidant (Turcotte and Saheb, 1978) and in addition, there are previous established reports of high antioxidant enzyme activity (Chi *et al*., 2015). The phytochemical screening of ethyl acetate extract of all the fungi, except *Rigidoporus vinctus* BT-GA2 and *Candida railenensis* BT-GAAB1, showed the presence of phenolic compounds, which was positively correlated with the antioxidant potential (Campos *et al*., 2015; Elleuch *et al*., 2010; Yadav *et al*., 2014).

### 3.5 Cytotoxic activity

**Fig. 4:**
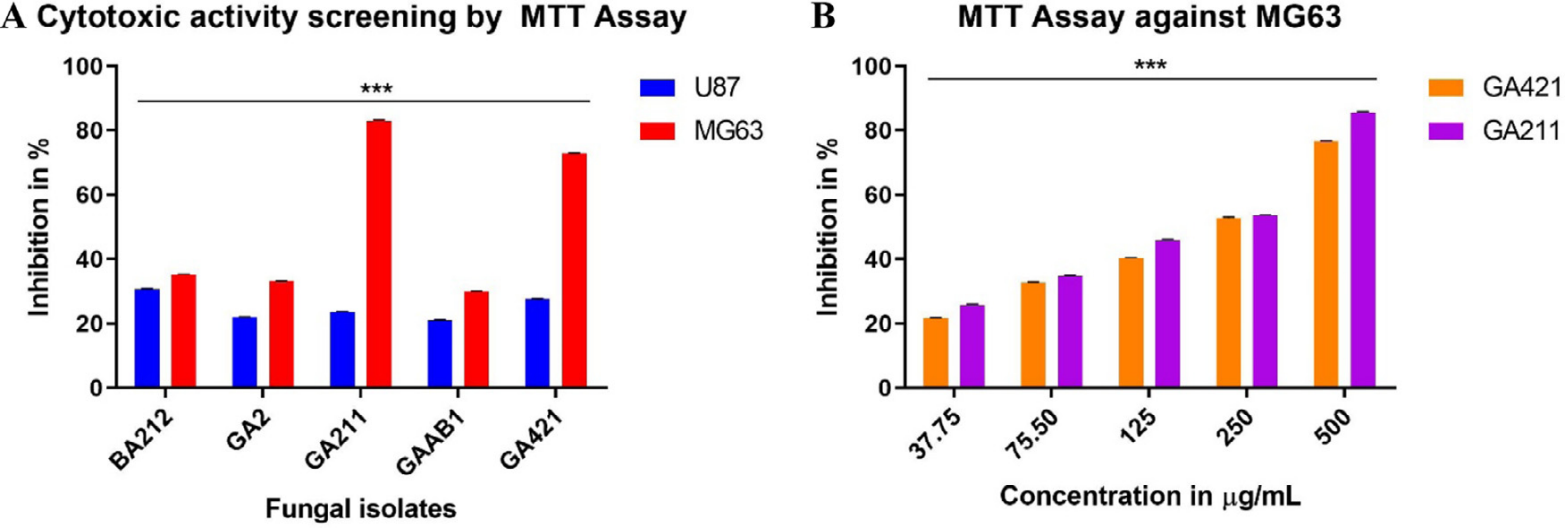
Cytotoxic activity screening and MTT Assay against MG63 cell line. A, Cytotoxic activity of all the fungal extracts, at a concentration of 500 μg/mL, were assessed against MG63 and U87 cell lines. B, the cytotoxic activity of fungal crude extracts was assessed using MTT Assay against MG63 cells, with different concentrations of crude fungal extracts ranging from 37.75 μg/mL to 500 μg/mL. The results were presented as the mean % inhibition (± SE, n=3). Statistical significance is represented by ***, p < 0.001.

The growth inhibitory effects of crude extracts were studied on human cancer cell lines MG63 and U87 using MTT assay. Both the cells were incubated with 500 μg/mL of crude extracts for 48h. No crude extract produced lower growth inhibition on U87 cells (20% - 30%), but all five crude extracts exhibited potent cytotoxic activity against MG63 cells with percentage inhibition ranging from (30% - 80%). With MG63 cells, the highest cytotoxic activity was displayed by *Pichia kudriavzevii* BT-GA211 (83% growth inhibition), followed by *Cystobasidium minutum* BT-GA421 (73%), while rest of the fungal crude extracts showed less than 35% inhibition. Two-way ANOVA followed by Tukey's pairwise comparison was conducted and the results revealed that all the crude extracts were significantly different between treatment groups ***p < 0.001. In addition, to evaluate the inhibitory concentration (IC_50_) of *Pichia kudriavzevii* BT-GA211 and *Cystobasidium minutum* BT-GA421 extracts, MG63 cells were incubated with their different concentrations, ranging from 37.75 to 500 μg/mL. As the concentration of the crude extract increased, *Pichia kudriavzevii* BT-GA211 displayed an increase in percentage of inhibition (from 26% to 86%), while in the case of *Cystobasidium minutum* BT-GA421, the increase in percentage of inhibition was limited (22% to 77%). In addition, *Pichia kudriavzevii* BT-GA211 exhibited a lower IC_50_ value of 145.1 ± 1.086 μg/mL, while *Cystobasidium minutum* BT-GA421 produced a higher IC_50_ value of 178.8 ± 1.049 μg/mL (Figure 4, Table 4). Paclitaxel (IC_50_ value = 0.042 ± 1.115 μg/mL) was used as positive control. Based on the calculated Pearson coefficient, both of them exhibited a very strong correlation (r = 0.9 – 0.99) between the cytotoxic activity against MG63 cell line and free radical scavenging properties using all the three assays. *Cystobasidium minutum* BT-GA421 exhibited a highest correlation between ABTS assay and MTT assay with an r value of 0.986, while *Pichia kudriavzevii* BT-GA211 exhibited the strongest correlation between FRAP Assay and MTT Assay with an r value of 0.945 (Figure 3D). Also, the correlations were found to be statistically significant (*p* < 0.01).

**Table 4:** Cytotoxic activity of fungal crude extracts against MG63 cells.

Cytotoxic IC_50_ of ethyl acetate extracts of fungal isolates against MG63 (Human osteosarcoma) cells	
IC_50_ (μg/mL)	
Isolates	MG63
BT-GA421	178.8 ± 1.049
BT-GA211	145.1 ± 1.086
Paclitaxel (Positive Control)	0.042 ± 1.115

*P. kudriavzevii* BT-GA211, along with *C. minutum* BT-GA421, exhibited potent cytotoxic activity against bone osteosarcoma cells. In 2017, Saber reported anticancer effect of metabolites secreted by *P. kudriavzevii* AS12 (also called benign probiotic yeast strain) and found that induction of apoptosis is the main mechanism of the anticancer effect (Saber *et al*., 2017). None of the extracts showed cytotoxic activity against glioblastoma cells. Glioblastoma is one of the most aggressive and chemo-resistant cancer (Da Ros *et al*., 2018) and this reveals the fact that most of compounds show either no or little activity against glioblastoma cells.

### 3.6 GC-MS analysis

The extracts were analysed by GC-MS, the mass spectrum of compounds was obtained by electron ionization and components identified by comparison of their mass spectral fragmentation patterns with that in National Institute of Standard and Technology (NIST14) library. The phytochemical constituents of the crude ethyl acetate extract of *Pichia kudriavzevii* BT-GA211, *Cystobasidium minutum* BT-GA421, *Grammothele fuligo* BT-BA212, *Candida railenensis* BT-GAAB1 and *Rigidoporus vinctus* BT-GA2 were identified and GC chromatogram, retention time, molecular weight, molecular formula was tabulated (Table 5). The phytocompounds Phenol,2,4-bis(1,1-dimethylethyl)-; Arsenous acid, tris(trimethylsilyl), ester; Silicic acid, diethyl bis(trimethylsilyl) ester and Butylated Hydroxytoluene were present in crude extract of more than one fungus.

**Table 5:** GC-MS analysis of fungal crude extracts.

List of metabolites present in Ethyl acetate extract of endophytic fungi						
BT-BA212						
S. I	R T (min.)	Compound	Molecular Formula	Molecular Weight	Activity	Mass Spectrum
1	13.272		C_14_H_22_O	206.32	Antioxidant, Anticancer, Antifungal, Antibacterial (Ren *et al*., 2019)	
2	28.675		C_15_H_1_N_2_	222.28	No Activity reported.	
3	30.072		C_20_H_21_N_2_O_5_	369.39	No Activity reported.	
4	30.398		C_22_H_42_O_4_	370.56	Antioxidant, antimicrobial, antiproliferative (Paramanantham and Murugesan, 2014)	
BT-GA2						
1	13.249		C_15_H_24_O	220.35	Antioxidant, Antimicrobial (Turcotte and Saheb, 1978)	
2	30.072		C_15_H_13_N	207.27	Antimicrobial, Antitumor (Vijayakumari and Raj, 2019)	
3	30.438		C_9_H_27_AsO_3_Si_3_	342.49	No Activity reported.	
BT-GA421						
1	13.295		C_14_H_22_O	206.32	Antioxidant, Anticancer, Antifungal, Antibacterial (Ren *et al*., 2019)	
2	22.925		C_20_H_30_O_4_	334	No activity reported.	
3	26.65		C_19_H_21_NOS	311.4	No activity reported.	
4	30.066		C_9_H_27_AsO_3_Si_3_	342.49	No Activity reported.	
5	30.421		C_10_H_28_O_4_Si_3_	296.58	Antibacterial, (Juliet *et al*., 2018)	
BT-GAAB1						
1	13.289		C_14_H_22_O	206.32	No Activity reported.	
2	30.066		C_15_H_13_N	207.27	Antimicrobial (Hussein *et al*., 2019)	
3	30.426		C_10_H_28_O_4_Si_3_	296.58	Antibacterial, (Juliet *et al*., 2018)	
BT-GA211						
1	13.255		C_15_H_24_O	220.35	Antioxidant, Antimicrobial (Turcotte and Saheb, 1978)	
2	30.066		C_18_H_45_AsO_3_Si_3_	871.302	No Activity reported.	

The development of pharmaceutical drug starts with unveiling the active component, biological assays to establish its activity profile and then followed by clinical studies to ascertain safety, efficacy and pharmacokinetic profile. GC-MS which comprises of Gas Chromatograph [GC] coupled with a Mass Spectrometer [MS] is an unanimously accepted method for the analysis of phyto-constituents. The GC-MS analysis of the crude extract of *Cystobasidium minutum* BT-GA421, *Grammothele fuligo* BT-BA212 and *Rigidoporus vinctus* BT-GA2 agrees with the observed bioactivity profile as indicated by the presence of Phenol,2,4-bis(1,1-dimethylethyl)-; Hexanedioic acid, bis (2-ethylhexyl) ester and Butylated Hydroxytoluene respectively, which have both antimicrobial and antioxidant activity (Paramanantham and Murugesan, 2014; Ren *et al*., 2019; Turcotte and Saheb, 1978). The presence of antimicrobial compounds, 2-Ethylacridine and Silicic acid, diethyl bis(trimethylsilyl) ester in GC chromatogram of *Rigidoporus vinctus* BT-GA2 and *Candida railenensis* BT-GAAB1 respectively, is in agreement with the activity profile observed in this study (Juliet *et al*., 2018; Turcotte and Saheb, 1978; Vijayakumari and Raj, 2019).

Apart from the secondary metabolites, these fungi have been extensively studied for their alternate products. For instance, alcohol dehydrogenase (RvADH) was isolated and characterized from *R. vinctus* with an optimum activity observed at pH 9.0 and inactivation half-life of 3.4 min. at 50°C (Ken *et al*., 2016). *C. minutu*m has been found to possess high carotene productivity (Dhaliwal and Chandra, 2015; Yurkov *et al*., 2015). Similarly, the ability of *G. fuligo* to secrete amylase enzymes and ligninolytic enzymes has been well documented. (Naidu *et al*., 2017; Prasher IB and Chauhan R, 2013). Even some of the isolated endophytic fungi and their products have significant application in commercial industries. Hexanedioic acid-bis-(2-ethylhexyl) ester, found in the crude extract of *G. fuligo* BA212, is a plasticizer derivative and is very rarely reported from natural sources (Elleuch *et al*., 2010). *C. minutum* was found to produce an exemplary fungal P450, CYP53B1, a benzoate‐*para*‐hydroxylase, capable of catalysing the stereo‐ and regio-specific hydroxylation of non‐activated C–H bonds (Theron *et al*., 2019). Recently, the ability of *P. kudriavzevii* in synthesis of ZnO nanoparticles through green method of synthesis was also explored (Moghaddam *et al*., 2017). Different strains of *P. kudriavzevii* possess potential probiotic activity and may be important in indigenous fermented food production (Greppi *et al*., 2017). The fermentation liquid of *R. vinctus* induces agarwood formation in *A. sinensis* tree (Chen *et al*., 2018). While all these articles portray either the potential of the organism as such or the enzymes produced by them, not much study has been conducted to unravel the properties of the secondary metabolites produced by them. Also, these five fungi were identified to be endophytic but have not been previously isolated from marine algae. The present work is the first of its kind to report the occurrence of these fungi from marine algae along with shedding light on their secondary metabolite profiles. Few of the crude extracts showed potent antimicrobial and antioxidant activity along with significant potential to be used as anticancer drugs and hence, needs to be further investigated. GC-MS analysis revealed a few compounds such as 1H-Indole,3-ethyl-2-(2-pyridyl)-; 2H-Benzo(d,E)isoquinoline-1,3(1H,3H)-dione, 2-(2-acetoxyethyl)-6-(4-morpholyl)-; Arsenous acid, tris(trimethylsilyl), ester; Thiocarbamic acid, N,N-dimethyl,S-1,3-diphenyl-2-butenyl-ester; Phenol, 2,5-bis(1,1-dimethylethyl)- and Tris(tert-butyldimethylsilyloxy)arsane, for which no bioactivity has been reported. Further research on these compounds has to be conducted to identify novel potential candidates.

## 4 Conclusion

In conclusion, marine algae of Kerala coastline are rich in endophytic fungal biodiversity as explored in the present study. These endophytes are emergent sources for novel therapeutic bioactive compounds. This study supported the evidence that many bioactive compounds produced by endophytes may have an ultimate application in industries. Further studies are in progress on purification of these crude extracts and structure elucidation of pure compounds followed by their yield enhancement to identify the potential therapeutic candidates, all of which warrant further investigations. However, more endophytic fungi need to be explored for novel bioactive compounds to add to the collection.

## CRediT authorship contribution statement

**Harikrishnan M:** Methodology, Formal analysis, Investigation, Writing – original draft, Visualization. **Saipriya P. P:** Investigation. **Prabha Prakash:** Investigation. **C. Jayabaskaran:** Conceptualization, Funding acquisition. **Sarita G. Bhat:** Conceptualization, Methodology, Writing – review & editing, Supervision, Project administration, Funding acquisition.

## Declaration of competing interest

The authors declare that they have no known competing financial interests or personal relationships that could have appeared to influence the work reported in this paper.

## Appendix Supplementary materials

Image, application 1
