# Supplementary material for: Multi-functional bioactive secondary metabolites derived from endophytic fungi of marine algal origin
Source: Curr Res Microb Sci. 2021 May 12;2:100037. doi: 10.1016/j.crmicr.2021.100037 (PMC8610299; doi:10.1016/j.crmicr.2021.100037)
Supplement: Supplementary file 1 [file mmc1.docx]

**Supplementary Information**

**Multi- functional cytotoxic Bioactive secondary metabolites derived from Endophytic Fungi of Marine Algal origin**

Harikrishnan M^a^, Saipriya P. P^a^, Prabha Prakash^a^, Dr. C. Jayabaskaran P^b^, Dr. Sarita G. Bhat^a*^


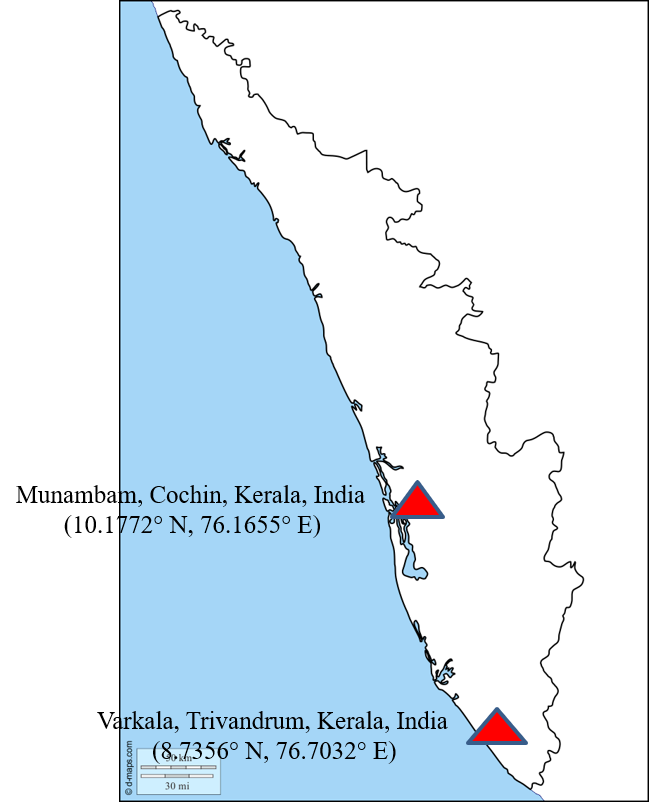


**Figure S1**: Sampling sites along the Kerala coastline. Image courtesy : www.d-maps.com (“Kerala free map from d-maps.com” )

*
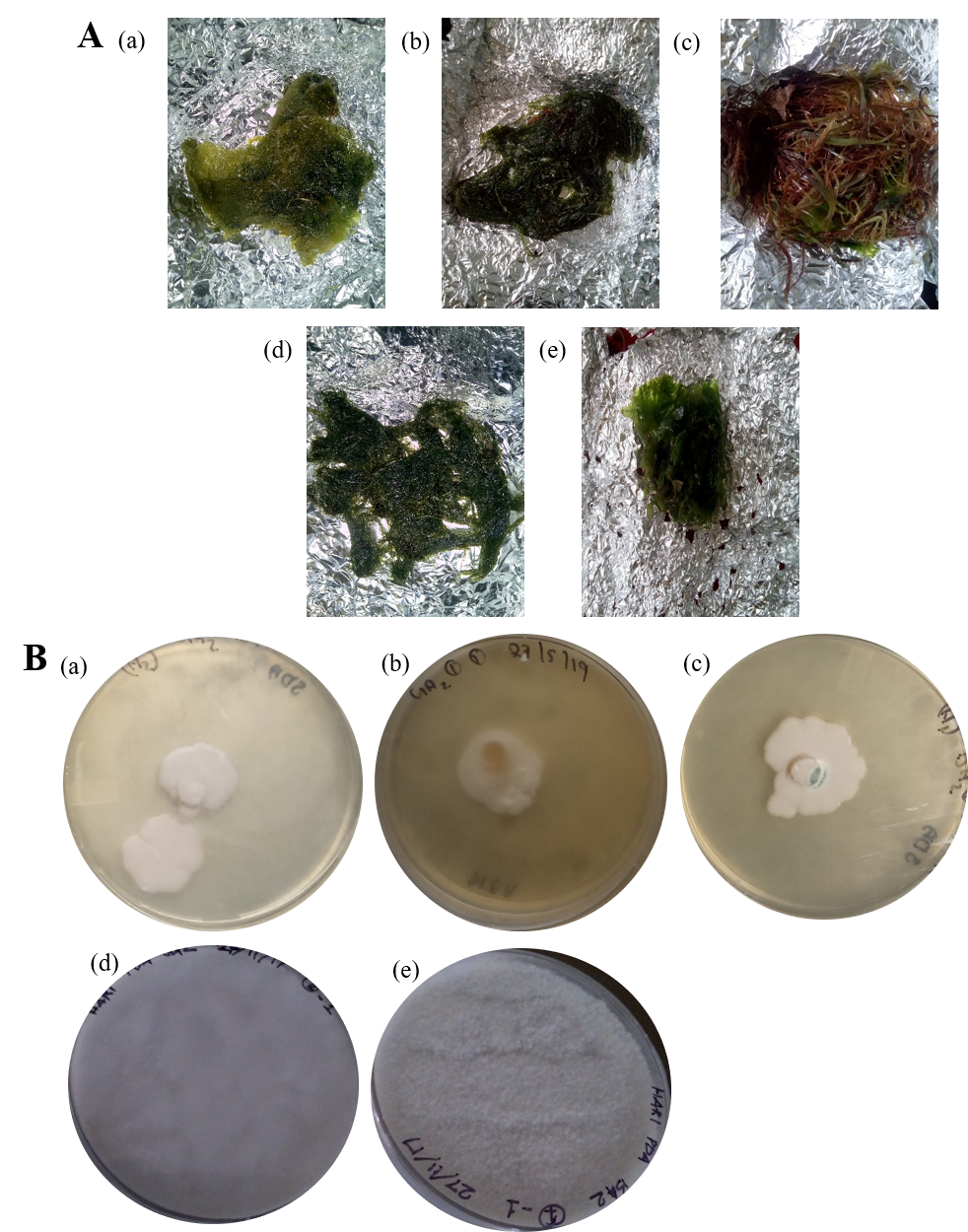
*

**Figure S2:** Marine algae sampled along the Kerala coastline and colony morphology of isolated endophytic fungi*. A, (a) Rhizoclonium, (b) Enteromorpha and (c) Undaria* obtained from Munambam site (10.1772° N, 76.1655° E), Cochin, Kerala, India*. (d) Chaetomorpha* *and (e) Ulva*

**Table S1**: Phytochemical screening of ethyl acetate extracts of fungal isolates.

| **Phytochemical screening of the ethyl acetate extracts of fungal isolate** | | | | |
| --- | --- | --- | --- | --- |
| **Isolates** | **Alkaloid** | **Phenolic** | **Terpenoid** | **Steroid** |
| BT-BA212 | + | + | - | + |
| BT-GA2 | + | - | + | + |
| BT-GA421 | + | + | - | - |
| BT-GAAB1 | - | + | + | - |
| BT-GA211 | + | + | - | - |

‘+’ indicates the presence of the compound, while ‘– ‘indicates the absence of the compound.

**Table S2**: Antibacterial activities (inhibitory zone assay) of ethyl acetate extract of fungal isolates.

| **Antibacterial activities (inhibitory zone assay) of ethyl acetate extract of fungal isolates** | | | | |
| --- | --- | --- | --- | --- |
| **Isolates** | ***P. aeruginosa*** | ***S. aureus*** | ***B. pumilus*** | ***E. coli*** |
| BT-BA212 | + | - | - | - |
| BT-GA2 | + | - | - | - |
| BT-GA421 | + | - | - | - |
| BT-GAAB1 | + | - | - | - |
| BT-GA211 | - | - | - | - |

‘+’ indicates the presence of the zone of inhibition ( sensitive), while ‘–‘ indicates the absence of zone of inhibition .
